# Vitamin D: beyond bone

**DOI:** 10.1111/nyas.12129

**Published:** 2013-05-17

**Authors:** Sylvia Christakos, Martin Hewison, David G Gardner, Carol L Wagner, Igor N Sergeev, Erica Rutten, Anastassios G Pittas, Ricardo Boland, Luigi Ferrucci, Daniel D Bikle

**Affiliations:** 1 Department of Biochemistry and Molecular Biology, UMDNJ-New Jersey Medical SchoolNewark, New Jersey; 2 Department of Orthopedic Surgery, The David Geffen School of Medicine at the University of CaliforniaLos Angeles, California; 3 Diabetes Center and Department of Medicine, University of CaliforniaSan Francisco, California; 4 Division of Neonatology, Department of Pediatrics, Medical University of South CarolinaCharleston, South Carolina; 5 Department of Health and Nutritional Sciences, South Dakota State UniversityBrookings, South Dakota; 6 Ciro, Center of Expertise for Chronic Organ FailureHorn, the Netherlands; 7 Division of Endocrinology, Diabetes and Metabolism, Tufts Medical CenterBoston, Massachusetts; 8 Departamento de Biología, Bioquímica, y Farmacia, Universidad Nacional del SurBahia Blanca, Argentina; 9 Longitudinal Studies Section, National Institute on Aging, National Institutes of HealthBaltimore, Maryland; 10 Departments of Medicine and Dermatology, University of California San Francisco, and VA Medical CenterSan Francisco, California

**Keywords:** vitamin D, cancer, immunity, pregnancy, obesity, diabetes, pulmonary disease, muscle, aging, cognitive function

## Abstract

In recent years, vitamin D has been received increased attention due to the resurgence of vitamin D deficiency and rickets in developed countries and the identification of extraskeletal effects of vitamin D, suggesting unexpected benefits of vitamin D in health and disease, beyond bone health. The possibility of extraskeletal effects of vitamin D was first noted with the discovery of the vitamin D receptor (VDR) in tissues and cells that are not involved in maintaining mineral homeostasis and bone health, including skin, placenta, pancreas, breast, prostate and colon cancer cells, and activated T cells. However, the biological significance of the expression of the VDR in different tissues is not fully understood, and the role of vitamin D in extraskeletal health has been a matter of debate. This report summarizes recent research on the roles for vitamin D in cancer, immunity and autoimmune diseases, cardiovascular and respiratory health, pregnancy, obesity, erythropoiesis, diabetes, muscle function, and aging.

## Introduction

Traditionally, vitamin D has been considered almost exclusively in the context of its role in calcium homeostasis. Vitamin D can either be taken in the diet, largely though fortification of dairy products, or synthesized in the skin from 7-dehydrocholesterol upon exposure to UV irradiation. Whether ingested or synthesized, vitamin D is transported to the liver, where it is hydroxylated at position 25 to form 25-hydroxyvitamin D (25(OH)D_2_ or 25(OH)D_3_), the major circulating form of vitamin D. Next, 25(OH)D_3_ is transported to the kidney, where it is hydroxylated at position 1 by the enzyme CYP27B1 to form 1,25-dihydroxyvitamin D (1,25(OH)_2_D_3_), the most active form of vitamin D, which is then transported to target tissues, where it functions like a steroid, binding to the vitamin D receptor (VDR). The VDR heterodimerizes with the retinoid X receptor (RXR), and the VDR/RXR complex binds to VDR-responsive elements found in or around target genes and, in association with various co-activators, results in the transcription of target genes. When there is a need to increase blood calcium levels (e.g., during growth or pregnancy), 1,25(OH)_2_D_3_ acts in the intestine to increase calcium absorption. If this increased intestinal absorption is insufficient to restore normal calcium levels, 1,25(OH)_2_D_3_ works in concert with the parathyroid hormone (PTH) in the kidney to promote calcium reabsorption from the distal tube, and in the skeletal system to release calcium from bones.

While it was long held that vitamin D acted only at the intestine, kidney, and skeleton, and that its function was limited to calcium homeostasis, the possibility of extraskeletal effects has been considered for decades as a result of the discovery of the VDR in tissues that have no involvement in calcium homeostasis (e.g., skin, placenta, pancreas, breast, prostate and colon cancer cells, and activated T cells). Discovery of the VDR and CYP27B1 in these tissues led to exploration of the roles and mechanisms of vitamin D function in each. On September 21, 2012, the Abbott Nutrition Health Institute and the Sackler Institute for Nutrition Science at the New York Academy of Sciences sponsored a conference, “Vitamin D: Beyond Bone,” that gathered researchers investigating vitamin D in a wide range of tissues and diseases.

## Nonclassical effects of vitamin D

### Vitamin D: beyond bone

Sylvia Christakos (UMDNJ-New Jersey Medical School) began the meeting by summarizing the metabolism and activity of vitamin D and introducing the emerging awareness of roles for vitamin D beyond calcium homeostasis and bone metabolism. Evidence in the laboratory, including the use of animal models, indicates that 1,25(OH)_2_D_3_ generates a number of extraskeletal effects, including inhibition of cancer progression, effects on the cardiovascular system and skin, modulation of innate immunity with subsequent killing of bacteria, and inhibition of certain autoimmune diseases (reviewed in Ref. 1). For example, in rats treated with the chemical carcinogen *N*-methyl *N*-nitrosourea (NMU), 1,25(OH)_2_D_3_ inhibits the progression of breast tumors. In addition, when 1,25(OH)_2_D_3_ is given prior to NMU, tumor incidence is prevented or reduced.^1^ 1,25(OH)_2_D_3_ also reduces the incidence and severity of prostate neoplasia in a mouse model (Ndx 3.1; *Pten* mutant mouse), and 1,25(OH)_2_D_3_ has tumor inhibitory activity in a mouse model of colorectal adenoma (Apc^min^). In order to determine mechanisms involved in inhibition of breast tumor growth, Christakos’ lab showed that C/EBPα, a transcription factor that has been shown to play a critical role in growth arrest of other cell types, is induced by 1,25(OH)_2_D_3_ in MCF-7 human breast cancer cells.^2^ C/EBPα was found to induce transcription of the vitamin D receptor in MCF-7 cells.^2^ Since the levels of the VDR correlate with the antiproliferative effects of 1,25(OH)_2_D_3_, and since it has been suggested that C/EBPα can be considered a potential tumor suppressor, these findings suggest mechanisms whereby 1,25(OH)_2_D_3_ may act to inhibit growth of breast cancer cells. These findings also identify C/EBPα as a 1,25(OH)_2_D_3_ target in breast cancer cells and provide evidence for C/EBPα as a candidate for breast cancer treatment.^2^

With regard to autoimmune diseases, 1,25(OH)_2_D_3_ has been shown to suppress type 1 diabetes in the non-obese diabetic (NOD) mouse model, to suppress experimental autoimmune encephalomyelitis (EAE) (a mouse model of multiple sclerosis (MS)), and to suppress mouse models of inflammatory bowel disease and systemic lupus erythematosus.^1^ Recent studies from Christakos’ lab have shown that inhibition of EAE is associated with inhibition of interleukin (IL)-17, a cytokine that plays a critical role in numerous inflammatory conditions and autoimmune diseases including MS. The mechanism of 1,25(OH)_2_D_3_ suppression of IL-17 was found to be transcriptional and to involve blocking of nuclear factor for activated T cells (NFAT, which is important for T cell receptor–mediated transcriptional regulation of IL-17), recruitment of histone deacetylase to the IL-17 promoter, and sequestration of Runt-related transcription factor 1 (Runx1) by the VDR.^3^ 1,25(OH)_2_D_3_ was also found to have a direct effect on the induction of Foxp3, a transcription factor that associates with NFAT and Runx1 for transcriptional repression.^3^ These results describe novel mechanisms and new concepts with regard to vitamin D and the immune system and suggest therapeutic targets for the control of autoimmune diseases.

**Figure 1:**
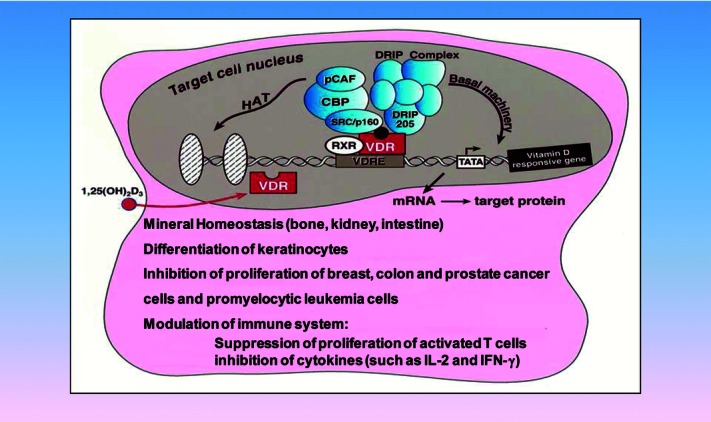
Genomic mechanism of vitamin D action. Mechanism of action of 1,25(OH)_2_D_3_ in target cells. The VDR heterodimerizes with the RXR. After interaction with the VDRE (vitamin D response element), transcription proceeds through the interaction of the VDR with coactivators and with the transcription machinery. The histone acetyltransferase (HAT) activity–containing complex (SRC/p160 and CBP), the DRIP complex, and additional coactivators not shown (including specific methyltransferases) are recruited by liganded VDR. 1,25(OH)_2_D_3_ is known to maintain calcium homeostasis and to affect numerous other cell types. Effects on other cell systems, including modulation of the immune system and inhibition of proliferation of cancer cells, are discussed. With permission from Christakos.^60^

Unlike the association between vitamin D deficiency and rickets, causal links between vitamin D deficiency and specific extraskeletal diseases have yet to be identified. However, the evidence in the laboratory of beneficial effects of 1,25(OH)_2_D_3_ beyond bone is compelling (summarized in Fig. 1). Findings in animal models may suggest mechanisms involving similar pathways in humans that could lead to the identification of new therapies.

### Vitamin D in immune function and disease prevention

Martin Hewison (the David Geffen School of Medicine, University of California) detailed one of the most prominent of the so-called nonclassical effects of vitamin D: its ability to act as a potent modulator of human immune responses. Evidence for this initially stemmed from two observations. First, many cells from both the innate and adaptive immune systems express the VDR. Second, antigen cells from the innate immune system, such as macrophages or dendritic cells (DCs), also express the vitamin D activation enzyme 1α-hydroxylase, also known as CYP27B1. As such, these cells are able to convert precursor 25(OH)D_3_, the major circulating form of vitamin D, to active 1,25(OH)_2_D_3_ that can then induce responses in the cells by binding to their VDRs and promoting transcriptional regulation.

This localized intracrine mechanism appears to be central to two key features of immune function: innate antibacterial activity and the presentation of antigen to cells from the adaptive immune system such as T lymphocytes (T cells). In macrophages and monocytes, cellular sensing of pathogens, such as *Mycobacterium tuberculosis*, by pattern recognition receptors, such as Toll-like receptors, enhances expression of CYP27B1 and the VDR, thereby increasing intracrine synthesis and activity of 1,25(OH)_2_D_3_.^4^ The resulting effects, including enhanced expression of the antibacterial proteins cathelicidin and β-defensin 2, and enhanced formation of autophagosomes, facilitate improved bacterial killing in a vitamin D–dependent fashion. Vitamin D insufficiency or deficiency may therefore impair innate immune responses and predispose individuals to infection, and low serum concentrations of 25(OH)D_3_ have been linked to infectious diseases such as tuberculosis. However, it is important to recognize that similar antibacterial responses to vitamin D have also been reported for other cell types within tissues, such as the skin, lungs, GI tract, and placenta, that may broadly be termed *barrier sites*. Thus, it is possible that vitamin D deficiency has a generalized detrimental impact on antibacterial responses and may therefore play a role in many types of infectious diseases.^5^ An important note by Hewison is that current data suggest that this particular immune response to vitamin D is restricted to primates, underlining a potential role for vitamin D in the evolution of primate immunity, but also limiting the scope of further *in vivo* studies to explore this activity.

The immunomodulatory effects of vitamin D also involve the adaptive immune system. Intracrine synthesis of 1,25(OH)_2_D_3_ by DCs decreases their maturation, thereby suppressing antigen presentation and decreasing T cell proliferation.^6^ Recent studies have shown that intracrine expression and activity of CYP27B1 by DCs is particularly effective in supporting the generation of tolerogenic regulatory T (T_reg_) cells, while simultaneously suppressing inflammatory IL-17–expressing T cells (Th17 cells).^7^ In this way, localized metabolism of 25(OH)D_3_ may play a pivotal role in prevention and/or treatment of autoimmune diseases, such as multiple sclerosis, rheumatoid arthritis, and inflammatory bowel disease. As with antibacterial responses to infection, it has been proposed that adaptive immune responses may be compromised in patients with low serum concentrations of 25(OH)D_3_, and association studies have shown links between autoimmune disease and vitamin D deficiency. The extent to which vitamin D deficiency is a cause of immune disease has yet to be demonstrated and will require new prospective clinical trials of vitamin D supplementation. Hewison stated that it will be important to define whether enhanced vitamin D–mediated immunity prevents immune disease or whether it can be used as therapy for these diseases. Moreover, it is possible that some diseases, such as leprosy and HIV infection, may be associated with dysregulation of the core vitamin D intracrine immune mechanism, and may therefore require higher circulating levels of 25(OH)D_3_ to reestablish normal immune responses. These and other facets of vitamin D and immune function will be important research challenges for the vitamin D community.

### Vitamin D in the heart and vascular system

David G. Gardner (University of California, San Francisco) expanded the discussion of nonclassical effects of vitamin D to include evidence of a significant role for vitamin D in the cardiovascular system. While the heart and vasculature have only recently been identified as potential targets of vitamin D action, a growing body of evidence suggests that the activated VDR plays an important role in regulating cardiovascular function.^8^ First, the VDR and the ligand-generating 25-hydroxyvitamin D_3_ 1-α-hydroxylase have been shown to be present in cardiac myocytes, cardiac fibroblasts, vascular smooth muscle cells, and vascular endothelial cells. Second, vitamin D deficiency in rodents has been shown to elicit increases in blood pressure and cardiac hypertrophy. Third, 1,25(OH)_2_D_3_ and its analogs have been shown to reverse agonist-induced myocyte hypertrophy *in vitro* and endogenous cardiac hypertrophy in the Dahl S rat, the spontaneous hypertensive rat (SHR), the spontaneous hypertensive heart failure–prone rat (SHR-HF), and the 5/6 nephrectomy model of chronic renal failure, *in vivo*. Fourth, a variety of cardiovascular disorders including congestive heart failure, coronary artery disease, and peripheral vascular disease have been linked to reduced circulating levels of 25(OH)D_3_. Fifth, and perhaps most important, a mouse with a complete deletion of the VDR gene demonstrates both hyperreninemic hypertension and cardiac hypertrophy.^9^

Gardner and colleagues examined the ability of paricalcitol, a bioactive analog of 1,25(OH)_2_D_3_, to prevent cardiac hypertrophy in rats infused with moderate doses of angiotensin II (800 ng/kg/min) over a 14-day period. Infusion of angiotensin II led to increased blood pressure, increased myocyte hypertrophy, increased expression of the hypertrophy-sensitive fetal gene program (i.e., atrial natriuretic peptide, B-type natriuretic peptide, and alpha skeletal actin gene expression), and increased cardiac interstitial fibrosis with augmented procollagen 1 and 3 expression. In each case, coadministration of paricalcitol (intraperitoneal injection of 300 ng/kg every 48 h), resulted in partial reversal of the angiotensin II effects.^10^

In an effort to define the role of the VDR in the myocardium, Gardner and colleagues created a mouse with a selective deletion of the fourth exon of the murine *Vdr* gene in cardiac myocytes. This mouse displayed an increase in myocyte size, left ventricular weight/body weight, and hypertrophy-dependent fetal gene expression compared with control littermates. There was no demonstrable increase in interstitial fibrosis. The mouse also demonstrated a reduction in end-diastolic and end-systolic volume by echocardiography and increased expression of modulatory calcineurin inhibitory protein 1 (MCIP1), a direct downstream target of calcineurin and NFAT that has been linked to the development of cardiac hypertrophy. Gardner's group showed that isoproterenol treatment of neonatal rat cardiac myocytes *in vitro* resulted in myocyte hypertrophy and increased MCIP1 expression. Coadministration of 1,25(OH)_2_D_3_ resulted in a dose-dependent reduction in MCIP1 expression.^11^

Vitamin D and the VDR have also been implicated in the support of normal endothelial function. Endothelial dysfunction, regarded by many as a harbinger of cardiovascular disease, has been linked to a number of disorders that are also associated with low circulating levels of 25(OH)D_3_. In some cases, treatment of these disorders with exogenous vitamin D or vitamin D metabolites has been shown to restore endothelial function. To establish a link between the VDR and endothelial function, Gardner and colleagues created a mouse with selective deletion of the *Vdr* gene in vascular endothelial cells. This mouse displays many of the phenotypic features normally associated with endothelial dysfunction.

Collectively, these data demonstrate that the vitamin D system—specifically, the ligand-bound VDR—plays an important role in the maintenance of cardiovascular health and suggest that vitamin D repletion may have important public health implications in controlling the prevalence of, and morbidity associated with, cardiovascular disease. Of equivalent importance, therapy with high potency vitamin D metabolites or their analogs may subserve an important role in the management of these diseases.

### Vitamin D during pregnancy and lactation

Vitamin D as a preprohormone has effects that extend beyond calcium metabolism and homeostasis throughout life but which are most vivid during pregnancy when vitamin D metabolism is disengaged from its usual constraints.^12^ At no other time during the lifecycle is 25(OH)D_3_ linked directly to 1,25(OH)_2_D_3_ production, the latter rising more than 2.5 times above nonpregnant levels.^12^ This elevation of 1,25(OH)_2_D_3_ likely serves the purpose of immune regulation, though there have been only a few studies that have evaluated the effect of vitamin D status on immune function during pregnancy to support this hypothesis. In addition, few randomized controlled trials have been conducted in pregnant women to determine optimal vitamin D status.^13^–^15^

Carol L. Wagner (Medical University of South Carolina) discussed two recent studies performed by her group in collaboration with Bruce Hollis's group—the National Institute of Child Health and Human Development (NICHD) Vitamin D Pregnancy Trial^12^ and the Thrasher Research Fund Vitamin D Community-Based Supplementation Trial^16^—that indicate that 400 IU vitamin D, the amount found in most prenatal vitamins, is inadequate and that 4000 IU/day is necessary to optimize 1,25(OH)_2_D_3_ production, achieved when total circulating 25(OH)D_3_ is at least 40 ng/mL (Fig. 1).^12^ When analyzed by maternal 25(OH)D_3_ concentration at various time points—i.e., baseline, by trimester, throughout pregnancy as mean, median, and area under the curve, or at delivery—the group taking 4000 IU/day attained optimal 1,25(OH)_2_D_3_ conversion from 25(OH)D_3_ throughout pregnancy at considerably higher rates than groups taking lower doses. Both studies provide evidence that 4000 IU/day is not only safe but is associated with fewer adverse events of pregnancy when taken together as comorbidities of pregnancy, compared with the lower dose groups.

Comorbidities of pregnancy appear to be directly linked to lower 25(OH)D_3_, and, conversely, increasing 25(OH)D_3_ appears to afford protection to the mother and developing fetus, with lower risk of preterm labor, preterm birth, or infection for each 10 ng/mL increase in total circulating 25(OH)D_3_ concentration.^16^ Such findings suggest a simple approach to reduce morbidities during pregnancy. Yet, before such findings are fully embraced, the mechanism(s) of action of vitamin D and its protective effect during pregnancy must be better delineated. Additional research that addresses the impact of vitamin D on immune homeostasis during pregnancy and its effects during early infancy and later childhood becomes essential.

These findings extend to lactation and early infancy. It was thought for decades that human milk was minimally sufficient in vitamin D;^17^ yet, preliminary results of the recently completed NICHD vitamin D supplementation trial during lactation show that when a mother is vitamin D replete, with an intake of 6400 IU vitamin D_3_/day, breast milk is replete and a breastfeeding infant has excellent vitamin D status without infant supplementation that is comparable to combined maternal and infant supplementation of 400 IU vitamin D_3_/day.^18^ The implications of safely achieving vitamin D sufficiency for both mother and breastfeeding infant solely through maternal supplementation is just beginning to be understood and will likely challenge researchers for decades to come.

### Vitamin D and obesity

Igor N. Sergeev (South Dakota State University) discussed the possibility of integrating vitamin D supplementation with current strategies in the prevention and treatment of obesity. The induction of adipocyte death through apoptosis is emerging as a promising strategy for addressing obesity.^19^,^20^ Increased adipose tissue mass is the result of both hypertrophy (an increase in adipocyte size) and hyperplasia (an increase in adipocyte number), and once adipocytes achieve a maximum size, further increase in adipose tissue mass involves an increase in adipocyte number. Thus, weight loss can result from not only a decrease in adipocyte size but also adipocyte number. Even a small increase in the amount of adipocyte apoptosis will prevent excessive accumulation of adipose tissue and can result in a significant loss of adipose tissue mass over time. Therefore, removal of adipocytes through apoptosis reduces body fat and can help in long-lasting maintenance of weight loss.

**Figure 2:**
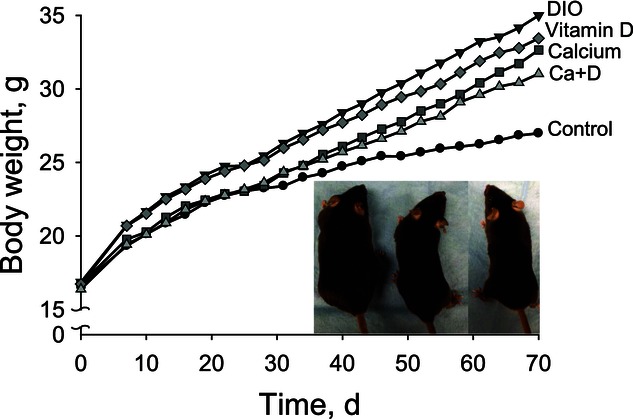
Body-weight gain in mice fed high-fat diets with increased levels of Ca and vitamin D. Weight matched mice were randomly assigned to the experimental high-fat (HF) diets containing 60% energy as fat (the normal control diet contained 10% energy as fat). The treatment groups on HF diet were high-calcium (Ca diet, 1.2% Ca), high-vitamin D_3_ (D diet; vitamin D_3_ intake 10 times higher than the recommended level of 1000 IU/kg) and high-Ca plus high-D_3_ (Ca + D diet). Data are means for each group and time point; *n* = 7–8 per group from week 1 to week 10. The insert shows (from left to right) mice from the DIO, control, and Ca + D groups at week 10. Data are unpublished and from work in progress.

The effects of the hormonal form of vitamin D, 1,25(OH)_2_D_3_, on apoptotic cell death are mediated via multiple signaling pathways that involve common regulators and effectors converging on cellular Ca^2+^.^19^,^21^ Sergeev and colleagues have shown that the critical characteristic of the apoptotic Ca^2+^ signal is a sustained increase in the concentration of intracellular Ca^2+^, reaching elevated but not cytotoxic levels.^22^ However, 1,25(OH)_2_D_3_-regulated, Ca^2+^-dependent apoptotic molecular targets have not been identified in adipose tissue. Thus, Sergeev and colleagues have been investigating the mechanism by which 1,25(OH)_2_D_3_ regulates apoptosis in adipocytes. Their results have demonstrated that 1,25(OH)_2_D_3_ induces, in a concentration- and time-dependent fashion, a sustained, prolonged increase in concentration of intracellular Ca^2+^ (the apoptotic Ca^2+^ signal) in mature mouse 3T3-L1 adipocytes.^23^ The 1,25(OH)_2_D_3_-induced increase in cellular Ca^2+^ is associated with activation of Ca^2+^-dependent μ-calpain and Ca^2+^/calpain-dependent caspase-12.^23^ Activation of these proteases is sufficient to effect morphological and biochemical changes consistent with apoptosis. The 1,25(OH)_2_D_3_-induced increase in cellular Ca^2+^ is also associated with reduced lipid accumulation in mature adipocytes.^23^ In prelimary but ongoing studies, a murine diet-induced obesity (DIO) model is being used to evaluate the role of vitamin D in adiposity. DIO mice (C57BL/6J) fed a high-vitamin D_3_ diet, in particular a high-vitamin D_3_ plus high-calcium diet, show decreased body and fat weight gain (Fig. 2; unpublished data) and improved markers of adiposity and vitamin D status (plasma concentrations of glucose, insulin, adiponectin, 25(OH)D_3_, 1,25(OH)_2_D_3_, and PTH), but an increased plasma Ca^2+^. High vitamin D_3_ and calcium intake is associated with activation of the Ca^2+^-dependent apoptotic proteases calpain and caspase-12 in adipose tissue of DIO mice. These preliminary findings suggest that the 1,25(OH)_2_D_3_-induced cellular Ca^2+^ signal can act as an apoptotic initiator that directly recruits Ca^2+^-dependent apoptotic effectors capable of executing apoptosis in adipose tissue. The preliminary results also suggest that high vitamin D and calcium intake decreases body and fat weight gain in diet-induced obesity, and that the potential mechanism of these effects involves activation of a Ca^2+^-mediated apoptotic pathway in adipose tissue (specifically, normalization of the activities of apoptotic proteases Ca^2+^-dependent calpain and Ca^2+^/calpain-dependent caspase-12 that are reduced in obesity). Targeting Ca^2+^ signaling and the vitamin D/Ca^2+^–dependent calpains and caspases in adipocytes with vitamin D supplementation may therefore be an effective and affordable approach for chemoprevention and treatment of obesity. Additional studies are warranted to evaluate this approach from a safety point of view and to identify the optimal levels of calcium and vitamin D intakes in obesity.

### Vitamin D and lung function in patients with chronic obstructive pulmonary disease

Erica Rutten (Ciro+, Centre of Expertise for Chronic Organ Failure, Netherlands) discussed her work investigating the association between plasma vitamin D levels and lung function in chronic obstructive pulmonary disease (COPD) patients. COPD is characterized by a persistent and, usually, progressive airflow limitation and is associated with an enhanced local inflammatory response. By 2030, COPD is predicted to become the fourth leading cause of death worldwide, and therefore accounts for about 8% of total deaths. Today, in addition to lung function impairment, COPD is recognized as a systemic disease with multiple comorbidities, such as osteoporosis, cardiovascular disease, renal impairment, and psychological disorders. In relation to the systemic pathology of COPD the role of vitamin D has generated interest, as vitamin D deficiency is often present in patients with COPD,^24^ dependent on the severity of COPD.^25^ Indeed, the prevalence of vitamin D deficiency, defined as plasma 25(OH) D_3_ concentrations below 20 ng/ml (50 nmol/L), was 58% in a cross-sectional study of 151 COPD patients entering pulmonary rehabilitation during summer.^26^ In the Bergen COPD Cohort Study, the prevalence of vitamin D deficiency was indeed higher in the COPD patients than in the control subjects, independent of season, age, smoking, comorbidity, and body mass index.^27^ Several potential explanations for the increased risk of vitamin D deficiency in patients with COPD include poor diet, less outdoor activity and thus less exposure to sunlight, accelerated skin aging due to smoking, renal dysfunction, and treatment with corticosteroids. Vitamin D is related to skeletal health in patients with COPD, and vitamin D deficiency is associated with low bone mineral density.^26^,^28^ Additionally, low vitamin D has been related to decreased exercise performance^26^,^29^ and exacerbation rate^30^ in COPD patients. And while vitamin D concentration has been related to lung function in the general population, the relation between lung function and vitamin D concentration has yet to be investigated in COPD patients only.

**Figure 3:**
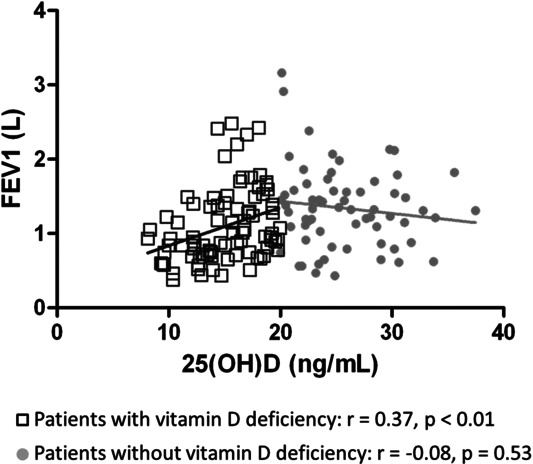
Lung function and plasma vitamin D levels in a group of 151 COPD patients. FEV1, forced expiratory volume in 1 s. These data are from an unpublished secondary analysis by Ruttan and colleagues.

Rutten and colleagues performed a secondary analysis of the publication by Romme *et al*.^26^ to investigate whether there is an independent association between lung function parameters and plasma vitamin D levels in a group of 151 COPD patients admitted for pulmonary rehabilitation at Ciro+. Plasma 25(OH)D_3_ levels, body mass index (BMI; weight in kg/(height in m)^2^), and lung function parameters (forced expiratory volume in 1s (FEV1), forced vital capacity (FVC), and diffusing capacity of the lung (DLCO)) were measured. The study group comprised 57% males, with mean age 65 ± 9 years, mean FEV1 47.2 ± 17.9% predicted, mean FVC 94.6 ± 20.6% predicted, and mean DLCO 53.2 ± 19.7% predicted; 58% of the patients were vitamin D deficient (vitamin D concentration <20 ng/ml). Pearson correlation coefficient showed significant correlations between plasma vitamin D concentration and FEV1 (*r* = 0.31, *P* < 0.01), FVC (*r* = 0.29, *P* < 0.01) and DLCO (*r* = 0.20, *P* = 0.01). After stratification for vitamin D deficiency, the correlations persisted only in the patients with vitamin D deficiency: FEV1 (*r* = 0.37, *P* < 0.01; Fig. 3, data are from an unpublished study), FVC (*r* = 0.25, *P* < 0.01), and DLCO (*r* = 0.32, *P* = 0.02). Multivariate regression analyses revealed that, after correction for age, gender, and BMI, vitamin D concentration remained independently associated with FEV1 (*β* = 0.26, *P* < 0.01) and DLCO (*β* = 0.25, *P* < 0.01), and there was a trend for FVC (*β* = 0.15, *P* < 0.08) in the subjects with vitamin D deficiency. From their unpublished secondary analysis, Rutten and colleagues conclude that there is evidence suggesting that vitamin D plays a role in the lung pathology of patients with COPD, which requires further investigation.

### Impact of cholecalciferol repletion on erythropoietin requirements in hemodialysis patients

Lily Li (Cleveland Clinic Lerner College of Medicine of Case Western Reserve University and Mount Sinai School of Medicine) presented pilot data from a randomized control trial investigating the impact of cholecalciferol repletion on erythropoietin requirements in vitamin D–deficient hemodialysis patients. Li mentioned that immune cells contain the machinery needed to convert 25(OH)D_3_ to 1,25(OH)_2_D_3_, and that there is evidence that this local production of 1,25(OH)_2_D_3_ has immunomodulatory properties. In a setting of decreased 25(OH)D_3_, the absence of 1,25(OH)_2_D_3_ production may enhance local inflammation.

Among patients with end-stage renal disease (ESRD) on hemodialysis, 25(OH)D_3_ deficiency is common, reported at greater than 80%.^32^ ESRD patients are commonly treated with 1,25(OH)_2_D_3_ for prevention of secondary hyperparathyroidism and bone loss. However, 25(OH)D_3_ is rarely repleted within this population, and the impact of 25(OH)D_3_ deficiency remains obscure. Anemia is also associated with ERSD and is the result of reduced erythropoietin production due to kidney disease as well as iron deficiency and inflammation-mediated abnormalities in iron metabolism. In this population, anemia not only causes impaired quality of life but also left ventricular hypertrophy, myocardial infarction, and increased mortality. Patients with ESRD-associated anemia are commonly treated with erythropoiesis-stimulating agents (ESA), which are very effective in the majority of the population. However, a subset of these patients develop erythropoietin hyporesponsiveness, which is thought to be caused by inadequate iron mobilization in the setting of inflammation, mediated in part by hepcidin, a small molecule produced by hepatocytes that is known to inhibit macrophage iron release and intestinal iron absorption. Since hepcidin production appears to be induced by IL-6, and 25(OH)D_3_ has been shown to suppress IL-6 production in monocytes,^33^ Li's group developed the hypothesis that in hemodialysis patients deficiency of 25(OH)D_3_, which is required for local production of 1,25(OH)_2_D_3_ in immune cells, leads to dysregulation of innate immunity and inflammation (e.g., enhanced IL-6 production), which alters iron metabolism and contributes to erythropoietin resistance.

They have initiated a randomized controlled trial of 25(OH)D_3_ repletion in vitamin D–deficient hemodialysis patients to evaluate efficacy and safety and the effects on inflammation and ESA requirements. Patients are given either no repletion (standard of care) or 50,000 IU/week of cholecalciferol for six weeks, or until 25(OH)D_3_ levels exceeded 35 ng/mL, and 10,000 IU/week afterwards. Their preliminary results show that 25(OH)D_3_ repletion was both safe and effective, with 25(OH)D_3_ levels increasing significantly in the treatment arm and no patients experiencing hypercalcemia or other adverse effects. In addition, patients in the treatment arm exhibit a decrease in ESA requirement at six months. Li and colleagues have isolated monocytes from patients and examined expression of a number of genes related to vitamin D metabolism and immune responses. Their observations include a decrease in IL-6 expression following 25(OH)D_3_ repletion at six months. These preliminary data indicate that 25(OH)D_3_ repletion treatment is safe, effective, and may result in lower ESA requirements, a result with significant economic implications.

## Translating epidemiological data into policy and clinical applications

### Vitamin D for diabetes

Anastassios G. Pittas (Tufts Medical Center) highlighted the potentially important role of vitamin D in a variety of non-skeletal medical conditions, including type 2 diabetes. To systemically appraise the available evidence on the role of vitamin D in diabetes, Pittas reviewed the original Bradford Hill criteria, which have been subsequently modified to include three general categories (mechanistic studies, direct evidence, and parallel evidence),^34^ and then applied these criteria to the available evidence.

From a biological perspective, the hypothesis that vitamin D may be a determinant of diabetes risk is plausible, as both impaired insulin secretion and action have been reported with vitamin D insufficiency. The effect of vitamin D may be direct, as supported by the expression of the VDR and the local production of 1,25(OH)_2_D_3_ in pancreatic β cells, or indirect via its role in regulating calcium homeostasis and calcium flux through cell membranes.

In humans, the role of vitamin D in type 2 diabetes is suggested by a large number of cross-sectional studies that have consistently found an inverse association between vitamin D status and prevalent hyperglycemia, including a reported seasonal variation in the control of glycemia in patients with diabetes, being worse in the winter when hypovitaminosis D is more prevalent.^35^ In the majority of longitudinal observational studies, lower vitamin D status is also associated with increased risk of incident type 2 diabetes. In the most recent systematic review of observational studies, Song *et al*.^36^ reported a 38% relative risk reduction in incident diabetes among individuals with the highest versus the lowest category of blood 25(OH)D_3_ concentration. In dose-response analyses, the risk of type 2 diabetes was reduced by 4% for each 4 ng/mL increment in blood 25(OH)D_3_ concentration.

However, Pittas cautioned that one should resist making inferences on the basis of observational studies because of the possibility of confounding issues, which may be especially true when assessing vitamin D status, because while 25(OH)D_3_ concentration is an excellent marker of good health many of the variables that contribute to 25(OH)D_3_ concentration (e.g., skin pigmentation, physical inactivity, aging, unhealthy dietary patterns) are also risk factors for the development of diabetes. Therefore, evidence from intervention studies—which is the most direct evidence—is critically important before one can conclude that vitamin D has a role in the prevention or treatment of diabetes. The results from small clinical trials and *post hoc* analyses of larger trials on the effect of vitamin D supplementation on diabetes-related parameters have been inconclusive, although vitamin D appears to have beneficial effects in persons at risk for diabetes.^36^–^39^ However, no firm conclusions can be drawn from the available intervention studies because of several limitations: (1) nearly all studies were underpowered; (2) the minority of studies were designed specifically for glycemic outcomes; (3) most studies did not report use of diabetes medications at baseline or during the study; (4) some trials used large infrequent doses of vitamin D, which may be metabolized differently compared to daily doses and may provide either no benefit or result in an unfavorable benefit/risk ratio.

Pittas concluded that although the observation studies strongly suggest an important role for vitamin D in type 2 diabetes, and such a role is biologically plausible, there is lack of evidence from intervention studies to support the contention that type 2 diabetes can be improved or prevented by raising 25(OH)D_3_ concentration. On numerous earlier occasions, encouraging findings from observational studies were not confirmed by well-designed clinical trials (e.g., hormone replacement therapy, β-carotene, vitamins C and E, and folic acid) and prevailing clinical practice was overturned. Therefore, confirmation of a potential beneficial effect of vitamin D in type 2 diabetes is needed in trials conducted in well-defined populations (e.g., pre-diabetes and early diabetes) specifically designed to test the compelling but yet unproven hypothesis that vitamin D status is a direct contributor to the pathogenesis of type 2 diabetes.

### Molecular aspects of the role of vitamin D in muscle

Ricardo Boland (Universidad Nacional del Sur, Argentina) discussed molecular aspects of the role of vitamin D in skeletal muscle health and function. This is a topic of clinical significance because myopathy characterized by proximal muscle weakness and atrophy is a common symptom in vitamin D deficiency states.^40^ The loss of muscular strength and a reduction in muscle mass increases the risk of falls and leads to fractures. It has been reported that administration of the hormonal vitamin D metabolite 1,25(OH)_2_D_3_ decreases the number of falls by improving muscle strength in elderly subjects.^41^ Consistent with this observation, there is evidence that 1,25(OH)_2_D_3_ regulates muscle growth and development and contractility.^40^ Moreover, the presence of the VDR in avian, murine, and human skeletal muscle has been demonstrated.^40^,^42^

As in other target cells, 1,25(OH)_2_D_3_ elicits long-term and short-term responses in skeletal muscle that involve genomic and non-genomic modes of actions, respectively. In the first, more classical mechanism, the hormone stimulates muscle cell proliferation and differentiation through nuclear VDR-mediated gene transcription, expressed by increased myoblast DNA synthesis followed by the induction of muscle specific myosin- and calcium-binding proteins. The non-genomic effects of 1,25(OH)_2_D_3_ are involved in the fast regulation of the calcium messenger system and growth-related signal transduction pathways in skeletal muscle cells. The hormone interacts with a membrane receptor, which leads to stimulation of adenylyl cyclase and phospholipases C, D, and A2 and activation of MAPK cascades.^43^ Boland pointed out that there is a wealth of evidence indicating that 1,25(OH)_2_D_3_ mainly acts in muscle through these membrane-initiated events and that the VDR plays a role in the activation of intracellular signaling pathways.^44^

Boland then focused his presentation on the signal transduction mechanisms of 1,25(OH)_2_D_3_ in skeletal muscle. 1,25(OH)_2_D_3_-induced transmembrane activation of adenylyl cyclase/cAMP/PKA and PLC/DAG plus IP_3_/PKC regulates Ca^2+^ influx mediated by voltage-dependent Ca^2+^ channels (VDDC).^43^ Boland´s group showed that, in muscle cells, the hormone also modulates Ca^2+^ influx through SOC channels that are activated by the PLC-mediated Ca^2+^ release from intracellular stores via IP_3_—a process known as capacitative calcium entry (CCE).^45^ Boland discussed the finding that 1,25(OH)_2_D_3_ rapidly induces reverse translocation of the VDR from the nucleus to the plasma membranes.^46^ In accordance with this observation, a complex is formed between the VDR and TRCP3, an integral protein of capacitative Ca^2+^ entry, suggesting an association between both proteins and a functional role of the VDR in 1,25(OH)_2_D_3_ activation of CCE. Further supporting this interpretation, transfection with a VDR antisense oligonucleotide inhibited Ca^2+^ influx through SOC channels. In some vertebrate systems, as in invertebrate photoreceptor cells, TRP proteins have been shown to interact with the PDZ domain–containing INAD proteins, which serve as acceptors for a molecule with a regulatory function, which Boland hypothesized could be the VDR. Of additional mechanistic importance, when muscle cells are treated with 1,25(OH)_2_D_3_ in the presence of a specific anti-INAD antibody or transfected with an anti-INAD antisense oligonucleotide, the hormone-dependent CCE was almost totally suppressed.^43^,^45^

These data enabled Boland and colleagues to propose a non-genomic mechanism by which 1,25(OH)_2_D_3_ regulates intracellular Ca^2+^ levels in skeletal muscle cells: by acting on voltage-dependent channels via protein kinase–mediated phosphorylation and on SOC/TRCP3 channels through VDR-joining membrane supramolecular complexes. Alterations in these mechanisms during vitamin D deficiency states may account for skeletal muscle weakness, in view of the key role that Ca^2+^ plays in the regulation of contractility.

In addition, 1,25(OH)_2_D_3_ modulates growth-related signal transduction pathways in skeletal muscle cells. It has been established that the hormone activates MAPKs (ERK1/2) and Akt. Of note, the formation of complexes between the VDR and Src is involved in the activation of these pathways by the hormone.^44^,^45^ Experimental data demonstrates that 1,25(OH)_2_D_3_ induces the translocation of activated ERK to the nucleus, where it phosphorylates the transcriptional factors Elk and CREB, which coregulate the expression of genes that mediate mitogenic effects of the hormone.^43^ Akt is activated by 1,25(OH)_2_D_3_ through PI3K, as the inhibitors Ly294002 and wortmannin block the increase in its phosphorylation in response to the hormone. Moreover, Akt mediates 1,25(OH)_2_D_3_-induced differentiation of skeletal muscle myoblasts to myotubes. With regard to the mechanism by which the hormone initiates its action at the muscle cell plasma membrane, preliminary work using MβCD and siRNA technology has shown that intact caveolae and caveolin-1 expression are involved in ERK1/2 and Akt activation by 1,25(OH)_2_D_3_ (C. Buitrago & R. Boland, submitted). Boland concluded that the information described contributes to the elucidation of the mechanisms involved in the regulation of contractility and myogenesis by 1,25(OH)_2_D_3_.

### Vitamin D and physical and cognitive function in older persons

Luigi Ferrucci (National Institute on Aging) discussed recent evidence linking vitamin D deficiency to the pathogenesis and clinical progression of chronic diseases highly prevalent in older persons. In large population-based studies, low levels of vitamin D predict loss of mobility and disability in basic activities of daily living, independent of age and other potential confounders.^47^ Based on these findings, some gerontologists claim that vitamin D has antiaging effects. Indeed, beyond its roles in bone health and calcium homeostasis, vitamin D affects many other physiological domains that are critically modified by aging. Unfortunately, the question of whether vitamin D prevents or slows aging cannot be easily tested. In spite of the intuitive nature of aging as a phenomenon, scientists have been unable to agree on good measures of biological aging because of great interindividual variability. Specific phenotypic changes occur in almost all aging individuals: changes in body composition, imbalance between production and utilization of energy, loss of redundancy of the homeostatic network, and loss of neurons and neuronal plasticity. Shifting attention from aging to the aging phenotype, the question of whether vitamin D affects major aging phenotypes becomes more approachable.

Data from the literature can generate some answers. Major changes in body composition that occur with aging include a decline in lean body mass (mostly muscle) and increase in fat mass. There is strong evidence that obesity is associated with poor vitamin D status due to fat sequestration.^48^ In fact, compared with the non-obese, higher doses of vitamin D are required in obese individuals. Also, regardless of dietary intake, weight loss results in increased serum 25(OH)D_3_ levels in overweight or obese women. However, some recent data suggest that low vitamin D predicts an accelerated increase in fat mass and incident obesity.

The importance of vitamin D for muscle has been discussed at length. Interestingly, VDR expression in human muscle tissue decreases with aging, and vitamin D level is strongly correlated with muscle quality and lower muscle fat infiltration. Consistently, in the Longitudinal Aging Study Amsterdam, low vitamin D and high parathyroid hormone were strong, independent risk factors for loss of muscle strength and muscle mass (sarcopenia).^49^ Low vitamin D levels also appear to be correlated with collagen deposition and are related to arterial stiffness.^50^ The role of vitamin D in energetics has been less thoroughly investigated. A recent study demonstrated that vitamin D level correlates with maximal oxygen consumption, and that supplementation of vitamin D increases fitness in athletes. Interestingly, a recent study found that VDRs are highly concentrated in the cristae of the mitochondria inner membrane.^51^ The role of vitamin D in homeostatic signaling is complex and pervasive. Vitamin D affects several pathways related to inflammation and cell proliferation, including inhibition of aromatase (conversion from testosterone to estrogens), COX2, prostaglandin receptor, NF-κB, HIF-1 and VEGF, and stimulation of IGFBP-3 and E-cadherin.^52^ In addition, poor vitamin D status is a risk factor for several autoimmune diseases, confirming the role of this vitamin in immune function.

The role of vitamin D in neurologic function and pathology has been studied from multiple perspectives. Vitamin D receptors are found in high concentration in various areas of the brain. Low vitamin D levels have been associated with accelerated cognitive decline in epidemiological studies, and low vitamin D dietary intake is associated with increased risk of Alzheimer's disease and depression.^53^,^54^ Mechanisms suggested for this association include β-amyloid formation/aggregation, dysregulation of GABAergic activity and NMDA receptor, and increased calcium entry into neurons. Overall, vitamin D appears to be implicated in many of the physiological and pathological changes that occur with aging. Whether vitamin D supplementation may positively impact the aging process remains unknown and will require further long-term intervention studies.

### Vitamin D dietary reference intakes

Daniel Bikle (University of California and VA Medical Center, San Francisco) addressed the recent Institute of Medicine (IOM) recommendations for dietary intake of vitamin D and evaluated whether they were sufficient in the context of emerging data about the roles of vitamin D and the prevalence of deficiency. Recommendations for the appropriate amount of vitamin D to be taken for optimal health—be it for musculoskeletal strength or for the numerous nonskeletal presumptive benefits of vitamin D—remain contentious. There is general consensus that the earlier recommendation of 400 IU of vitamin D/day was insufficient. Recently an IOM expert panel established to formulate new guidelines recommended that 600 IU/day would suffice for the general public aged 1–70 years, with the dose increased to 800 IU/day for those older than 70 years. However, doses up to 4000 IU/day were considered safe.^55^ The task force compiling the Endocrine Society Clinical Practice Guidelines (ESCPG) reached similar conclusions.^56^ Vitamin D status is best assessed by the circulating levels of 25(OH)D_3_. There is considerable variation among individuals with regard to the serum level of 25(OH)D_3_ achieved with a given dose of oral vitamin D, and it is the serum level that counts with respect to biologic effect. The IOM expert panel concluded that most Americans (97.5%) had adequate levels of vitamin D, based on their conclusion that a 25(OH)D_3_ of 20 ng/mL or greater was sufficient, raising the question of whether supplementation was even needed.

This conclusion was based on data from the 2001–2004 NHANES survey^57^ that primarily sampled Caucasians, with limited sampling in Northern latitudes during the winter, the time and region that people would most likely have lower vitamin D levels. In that survey the average 25(OH)D_3_ level was over 20 ng/mL. People of color, especially African-Americans, have substantially lower levels of 25(OH)D_3_. Moreover, a number of studies have shown that prevailing levels of 25(OH)D_3_, especially in older subjects, the institutionalized, and in most other countries, are well below those documented in the NHANES survey.^58^ As parts of the aging process, the skin becomes less able to make vitamin D for a given amount of UVB exposure, the kidneys become less able to produce the active metabolite 1,25(OH_2_)D_3_, and the intestine becomes less able to respond to the 1,25(OH_2_)D_3_ with respect to calcium absorption. Furthermore, the conclusion by the IOM expert panel, that 20 ng/mL is sufficient, differs from that reached by the task force compiling the ESCPG. This task force recommended a higher target level, namely 30 ng/mL (75 mM), and pointed out that many individuals with a variety of conditions, including obesity, dark skin, malabsorption, renal failure, lactose intolerance, ingestion of certain drugs, and the institutionalized, are likely not to achieve adequate 25(OH)D levels by ingesting only 600 IU/day. If 30 ng/mL is deemed the optimal level (and neither set of recommendations considers this level unsafe), then the percent of Americans that maintain the optimal level falls well below 50%.

A further complication in trying to achieve a given level of 25(OH)D_3_ is that 25(OH)D_3_ assays have not proven consistent, making decisions regarding supplementation all the more difficult. This situation is improving with the availability of standards from the National Institute of Standards and Technology. However, those assays relying on binding proteins or antibodies tend to report lower 25(OH)D levels than assays employing chromatography/mass spectroscopy.^59^ Moreover, the latter can separately determine 25(OH)D_2_ and 25(OH)D_3_ levels, as well as the level of 3-epimer of 25(OH)D_3_, capabilities that are lacking in the binding protein/antibody assays. While the clinical significance of these differences in assays is not clear, they do complicate decision making when a fixed target level of 25(OH)D_3_ is being sought. In practical terms, young, healthy, light-skinned individuals with good diets that include dairy products and who have adequate exposure to sunlight are not likely to be vitamin D deficient or need to be tested. Individuals of color, the aged, those with gastrointestinal disorders, the institutionalized, and those with poor nutrition should be tested and treated appropriately to achieve a 25(OH)D_3_ level of at least 20 ng/mL, or perhaps closer to 30 ng/mL. Daily doses appear to be safer and more efficacious than large doses administered once or twice a year.

## Conclusions

The conference “Vitamin D: Beyond Bone” explo-red a considerable range of functions for vitamin D and potential therapeutic implications outside of calcium homeostasis and bone health, highlighting the increased understanding of vitamin D, as well as how much is yet to be learned. The scope of vitamin D research and its therapeutic possibilities, as presented by researchers across many different disciplines, generated enthusiasm tempered with some skepticism. Continuing research is needed to better understand the relationships between vitamin D and extraskeletal health and to determine the optimal dose of vitamin D for individuals based on age, health conditions, and other factors.
